# P-1312. MPox Seroprevalence and Seroincidence among People who Inject Drugs in the Mexico-U.S. Border Region

**DOI:** 10.1093/ofid/ofae631.1493

**Published:** 2025-01-29

**Authors:** Anne Rimoin, Megan Halbrook, Irina Artamanova, Subbian Satheshkumar Panayampalli, Michael Townsend, Lalita Priyamvada, Will Carson, Daniela Abramovitz, Antoine Chaillon, David Smith, Alicia Harvey-Vera, Carlos F Vera, Britt Skaathun, Steffanie Strathdee, Nabojeet Das, Gudelia Rangel

**Affiliations:** UCLA, Los Angeles, California; UCLA, Los Angeles, California; UCSD, San Diego, California; Centers for Disease Control and Prevention, Atlanta, Georgia; CDC, Atlanta, Georgia; CDC, Atlanta, Georgia; CDC, Atlanta, Georgia; UCSD, San Diego, California; UCSD, San Diego, California; University of California, San Diego, San Diego, California; UCSD, San Diego, California; UCSD, San Diego, California; University of California San Diego, La Jolla, California; UC San Diego, La Jolla, California; CDC, Atlanta, Georgia; UCSD, San Diego, California

## Abstract

**Background:**

We assessed seroprevalence and seroincidence against orthopoxvirus (OPXV) among people who inject drugs (PWID) in the U.S./Mexico border region during the COVID-19 pandemic.

**Methods:**

Between 09/21-11/23, PWID who lived in either San Diego (SD; N=129) or Tijuana (TJ; N=343) underwent a baseline and follow-up visit involving interviewer-administered surveys and venipuncture. Sera were batch tested at the U.S. Center for Disease Control and Prevention using an OPXV generic IgG ELISA using JYNNEOS^®^ vaccine as the viral antigen. Optical densities (OD) ≥0.1 were conservatively considered seropositive. Mpox seroprevalence was assessed after excluding known sources of anti-OPXV antibodies

**Results:**

Of 472 participants enrolled, median age was 45 (IQR:38-53), 72.9% were male (3.2% of whom reported sex with men), 11.3% had HIV and 25.8% were unhoused. Although no symptoms consistent with Mpox were reported during the study, 50 (10.6%) had seropositive results at baseline, follow-up or both. Baseline OPXV seroprevalence was 19.4% in SD and 3.2% in TJ, and Mpox seroincidence was 8.97 (95%CI:4.09-13.85) per 100 person-years in TJ vs. 1.81 (95%CI: 0.00-5.35) in SD, with a median of 5.8 months of follow-up (IQR: 4.53 – 6.31 months). Excluding participants who reported Mpox vaccination (N=3), smallpox vaccination (N=73), or those born prior to 1971 who could have received smallpox vaccination (N=140), likely Mpox seroprevalence was 7.8% (23/296), with 13/23 seronegative at baseline but seropositive at follow-up. Of these 13 seroconveters, 5 were female of whom 2 were HIV+.

**Conclusion:**

These preliminary data suggest a undetected Mpox circulation in the Mexico-U.S. border region between 2022 and 2023 and that asymptomatic or mildly symptomatic Mpox infection may be more common than previously recognized. Although the clinical implications of these findings remain unclear, expansion of Mpox vaccination to PWID may be warranted.

**Disclosures:**

**David Smith, MD, MAS**, Bayer: Advisor/Consultant|Gilead: Advisor/Consultant|Model Medicines: Advisor/Consultant|Red Queen Therapuetics: Advisor/Consultant

